# Effectiveness of steam sterilization of reusable medical devices in primary and secondary care public hospitals in Nepal and factors associated with ineffective sterilization: A nation-wide cross-sectional study

**DOI:** 10.1371/journal.pone.0225595

**Published:** 2019-11-21

**Authors:** Gopal Panta, Ann K. Richardson, Ian C. Shaw, Stephen Chambers, Patricia A. Coope

**Affiliations:** 1 School of Health Sciences, University of Canterbury, Christchurch, New Zealand; 2 School of Physical and Chemical Sciences, University of Canterbury, Christchurch, New Zealand; 3 Department of Pathology and Biomedical Science, University of Otago, Christchurch, New Zealand; 4 College of Education, Health and Human Development, University of Canterbury, Christchurch, New Zealand; FDA, UNITED STATES

## Abstract

**Background:**

Inadequate sterilization of reusable medical devices can lead to healthcare associated infections (HAIs) through person-to-person or environmental transmission of pathogens. Autoclaving (steam sterilization) is most commonly used for sterilizing medical devices in healthcare facilities. We conducted a nation-wide cross-sectional study to evaluate the effectiveness of steam sterilization practices in primary and secondary care public hospitals in Nepal and to identify factors associated with ineffective sterilization.

**Methods:**

Using a stratified clustered random sampling, 13 primary- and secondary-care public hospitals in Nepal were selected. 189 steam sterilization cycles from these hospitals were evaluated for their effectiveness using self-contained biological indicators, class-5 chemical indicators, autoclave indicator tape and physical parameters. Information about the hospitals and the types of autoclaves being used was also collected. Data were analysed to estimate the proportion of ineffective steam sterilization cycles. Logistic regression was used to identify factors associated with ineffective sterilization.

**Findings:**

In primary and secondary care public hospitals in Nepal, 71.0% (95% CI 46.8% - 87.2%) of the autoclave cycles were ineffective (i.e. showed positive results) when tested with biological indicators and 69.8% (95% CI 44.4% - 87.0%) showed ‘reject’ results with class 5 chemical indicators. There was no statistically significant difference in proportions showing positive or reject results by hospital types for either biological (p = 0.51) or class 5 chemical (p = 0.87) indicators. Autoclave type and pressure achieved during sterilization were statistically significantly associated with steam sterilization failures, adjusted for holding period, evenness of pressure and barrier system used.

**Conclusion:**

Primary and secondary care hospitals in Nepal have a high proportion of steam sterilization failure, indicating a risk of person-to-person transmission of pathogens through reusable medical devices. There is an urgent need to improve steam sterilization processes in these hospitals.

## Introduction

Some medical devices are designed to be reused several times following adequate decontamination and reprocessing. Reusable medical devices are categorized as critical, semi-critical and non-critical [1]. Critical medical devices come in contact with sterile parts of the human body presenting a higher risk of infection to patients and sterilization is recommended for decontaminating such medical devices [2]. Effective sterilization ensures a medical device is free from viable microorganisms, including spores, and represents the highest level of decontamination. Steam sterilization, also known as autoclaving or moist-heat sterilization, uses saturated steam at a high temperature (usually 121°C) and is considered the most robust, most common and cost-effective sterilization method [3,4].

Inadequate sterilization of medical devices carries a risk of infections through person-to-person and environmental transmission of pathogens [5]. Multiple studies have reported infections associated with inadequately reprocessed medical devices [6–10]. Despite this, the contribution of poor sterilization to healthcare associated infections (HAIs) globally or in resource-poor settings where reuse of medical devices is likely to be less standardized and regulated, has been under-reported. Inadequate sterilization of devices is one of several factors that contribute to HAIs, which are more common in low- and middle-income countries (pooled prevalence of affected patients = 10.2%; 95% CI 9.0% - 13.0%) than in high-income countries (pooled prevalence of affected patients = 7.1 per 100 patients; 95% CI 6.5–7.8), with surgical site infection (SSI) being the most frequent type of HAI [5,11]. Tertiary care hospitals in Nepal have reported high rates of SSI (2.7 to 23.0 per 100 patients) but have not investigated a link between these infections and sterilization procedures [12–15].

Ensuring sterility of reusable medical devices in hospitals is a basic but critical component of infection prevention and control [5], and its importance has increased with the increased use of surgery (including in primary care facilities) and increasing antimicrobial resistance. Ineffective steam sterilization practices in healthcare facilities in different parts of the world have been reported, but most reports are from dental practices, which might not be relevant to general healthcare facilities [16–30]. The reasons for ineffective sterilization practices are unclear and need to be explored in order to formulate interventions to improve reprocessing and reuse of medical devices. This study assesses the effectiveness of steam sterilization in primary (district-level and district hospitals) and secondary care (zonal hospitals) hospitals in Nepal, and identify factors associated with steam sterilization failures in these hospitals. This study also reports sensitivity and specificity of the class 5 chemical indicator against the biological indicator (gold standard) in detecting steam sterilization failures in public healthcare settings of Nepal.

## Methods

### Study design

This was a cross-sectional study where the study units were steam sterilization (autoclaving) cycles in hospitals in Nepal. There are 10 zonal, 62 district and 16 district-level hospitals in Nepal [31]. A stratified design with three strata (hospital types) with hospitals sampled from within each stratum using simple proportional allocation of hospitals was used. Each hospital represented a cluster of observations (the repeated sampling of the sterilization cycle).

We firstly considered a ‘reasonable’ estimate of required observations, assuming simple random sampling of units. This sample size was then adjusted for sampling clusters of observations. The key drivers of the sample size were the margin of error required and the assumption about the impact of clustering, measured by ‘rho’ (the intra-class correlation coefficient), which gave the calculation of the Design Effect (DEFF). The DEFF gave the factor by which the number of units of a simple random sample could be decreased while maintaining the same precision as the realized cluster sample [32]. With rho = 0.2 for each category of hospital and 95% confidence, the sample size of 189 was estimated for the stratified clustered design with a margin of error of 0.09 (Table 1).

**Table 1:** Sample sizes for assessing effectiveness of autoclave cycles in different hospital categories.

Hospital type	Number of hospitals	Randomly sampled hospitals	Consecutive autoclave cycles tested in each hospital	Autoclave cycles tested in each hospital category
Zonal hospital *(Secondary care hospital)*	10	2	12	24
District hospital *(Primary care hospital)*	62	9	15	135
District-level hospital *(Primary care hospital but smaller than district hospital)*	16	2	15	30
**Total number of autoclave cycles tested for effectiveness**				**189**

### Sample selection

Within each hospital type a simple random sample was taken to select the hospitals. For district hospitals, we wanted the sample (nine hospitals) spread across the seven provinces, so systematic random sampling was used. Within a hospital, autoclaving is a continuous process and the ‘population’ for the purpose of this study (i.e. total number of autoclave cycles) was effectively infinite. It was not practically possible to select the autoclave cycles randomly from such a population. Therefore, we tested a predetermined number of consecutive autoclave cycles in each hospital.

### Procedures

Basic information about each hospital was collected on a summary information sheet. Such information included number of beds, number of staff, available clinical services, decontamination activities performed, number of autoclaves, and autoclave types. Required information was obtained from the staff working in the relevant sections in the hospital or by observation.

The researcher labelled a ProSpore 2 Self-Contained Biological Indicator (Mesa Labs, Inc.; Catalog Number PS2-3-6-50) containing 10^6^ spores of *Geobacillus stearothermophilus* and a ProChem SSW Steam Sterilization Integrator (Mesa Labs, Inc.; Catalog Number CI-SSW)—a class 5 chemical indicator, with the same observation code [33,34]. Then the autoclave operator of the hospital packaged both the indicators together in the same way as the actual medical devices were packaged and prepared for a particular autoclave cycle, using the same wrapping material as that used for medical devices. An autoclave tape (Mesa Labs, Inc.; Catalog Number: CI-STP) was also affixed to the package containing the indicators [35]. The package with the indicators was then placed inside the autoclave with the packages of medical devices to be sterilized. The medical devices along with the indicators were autoclaved by the autoclave operator according to in-house procedures. The researcher read the pressure gauge of the autoclave chamber every minute, during the autoclave cycle, and recorded the pressures. The same process was used for all 189 autoclaving processes. It took multiple days to test the predetermined number of consecutive autoclave cycles in a hospital (Table 1) depending on the frequency of autoclave cycles at each hospital.

After completion of the autoclave cycle, the indicator package was retrieved from the autoclave chamber. The autoclave tape was checked for any change in colour. The package of indicators was opened and the ProChem SSW Steam Sterilization Integrator (class 5 chemical indicator) was checked to see whether the dark bar had entered the accept window. The biological indicator was taken out of the package, sealed, allowed to cool and then crushed according to the manufacturer’s instructions. Then, the tube was incubated at 57°C for 24 h along with an additional control tube (unexposed to sterilization cycle) in a portable incubator (Mesa Labs, Inc.; Model 1450). Following this, the tubes were examined to observe any change in colour. If the tube exposed to sterilization exhibited a colour change to or toward yellow (positive test result), the sterilization cycle was considered failed or ineffective. If the tube did not change colour (negative test result), the cycles were considered successful or effective. For the test to be valid, the control tube changed in colour to or towards yellow. The results of all the three indicators were recorded on a results form.

### Data analysis

Information from the study forms was entered into an Excel spreadsheet every day. After completion of field work, data in the spreadsheets were imported into *IBM SPSS Statistics 24* software. Imported data sets were checked for any errors and discrepancies, which were then corrected by referring to the study forms. Hospital variables such as hospital type, number of beds, and number of staff in different categories were tabulated. We performed descriptive analyses of chemical and biological test results, autoclave types, lengths of autoclave cycles, holding periods, maintenance of pressure during holding periods, and barrier systems used. The analysis included calculation of proportions and assessment of associations between variables. A logistic regression model for complex samples was used to identify factors associated with steam sterilization failures.

### Ethical considerations

Ethical approval for this study was obtained from the Ethical Review Board of the Nepal Health Research Council (Reg. No. 13/2016) and the Human Ethics Committee of the University of Canterbury (HEC 2015/139). Written consents were obtained from the medical superintendents or the incharges of the hospitals participating in this study.

## Results

### Characteristics of hospitals included in the study

Some characteristics including number of beds, total number of staff, space allocated for medical device reprocessing, number of available autoclaves and sterilization cycles per week varied across the hospitals (Table 2). However, other characteristics including staff allocated for medical device reprocessing, autoclave type, availability of procedure manual or flow charts, monitoring of sterilization cycles, maintenance of autoclaves and availability of spare parts were similar across the hospitals (Table 2). Of the thirteen hospitals, six (46%) had a separate area dedicated to reprocessing of medical devices. Decontamination activities performed in the hospitals included cleaning, chemical disinfection, boiling, steaming and autoclaving. Autoclaving was performed in all the hospitals for sterilizing critical medical devices, with the number of autoclaves per hospital ranging from one to three. Of the 24 autoclaves used at the hospitals, three were downward (gravity) displacement autoclaves and 21 were basic pressure-cooker type autoclaves.

**Table 2:** Characteristics of the hospitals selected in the study.

Hospital Type	Hospital code	Number of beds	Total staff	Staff allocated for medical device reprocessing	Dedicated space for sterilization	Autoclave type and number*	Autoclave cycles per week (approximate)	Procedure manuals or flow charts	Monitoring sterilization cycles with chemical/ biological indicators	Maintenance records	Spare parts
Zonal Hospitals	02	150	189	2	Yes	Upward displacement (2); Downward displacement (1)	20	Yes	No	No	No
	08	332	412	3	Yes	Downward displacement (2)	21	No	No	No	No
District Hospitals	01	15	29	2	Yes	Upward displacement (2)	7	No	No	No	No
	03	15	44	2	No	Upward displacement (1)	13	No	No	No	No
	04	60	67	2	Yes	Upward displacement (1)	18	No	No	No	No
	06	36	61	2	No	Upward displacement (3)	13	No	No	No	Yes
	07	50	58	2	No	Upward displacement (1)	15	No	No	No	No
	09	15	32	2	No	Upward displacement (3)	12	No	No	No	No
	11	25	44	2	No	Upward displacement (1)	13	No	No	No	No
	12	37	62	2	Yes	Upward displacement (3)	18	Yes	No	No	No
	13	31	53	2	Yes	Upward displacement (2)	15	No	No	No	No
District-level Hospitals	05	5	25	1	No	Upward displacement (1)	13	No	No	No	No
	10	4	14	1	No	Upward displacement (1)	12	No	No	No	No

* the numbers in parentheses indicate number of autoclaves

All thirteen hospitals provided inpatient and outpatient services, minor surgical services, family planning, immunisation, antenatal services, maternity services and laboratory services. Major surgical services (requiring an operating theatre) were also provided by all except two district hospitals and two district-level hospitals. Dental services were provided by all except the district-level hospitals.

### Sterilization pressures and holding periods

Pressures could not be recorded for 15.5% (95% CI 4.0% - 44.9%) of the sterilization cycles as four of the autoclaves had faulty pressure gauges. For the remaining sterilization cycles, pressures achieved inside the autoclaves during the holding periods varied from <10 psi to ≥15 psi (Table 3).

**Table 3:** Pressures achieved and maintenance of pressure during the holding periods of sterilization cycles.

Holding period pressure	Estimated Proportion	Standard Error	95% Confidence Interval	
			Lower	Upper
**Pressure achieved (n = 189)**				
Could not be recorded	15.5%	8.8%	4.0%	44.9%
≥15 psi	45.9%	11.0%	24.1%	69.4%
≥10 psi and<15 psi	27.6%	3.9%	19.9%	37.1%
<10 psi	10.9%	6.0%	3.0%	32.8%
**Maintenance of pressure (n = 160)**				
Continuous (plateau)	73.2%	12.5%	39.9%	91.8%
Intermittent (uneven)	26.8%	12.5%	8.2%	60.1%

The mean length of an autoclave cycle (the time period between the start and end of the sterilization cycle) was approximately 64 min (95% CI 55.8–72.56), and the mean holding period was 20 min (95% CI 14.3–25.7). The holding periods of autoclave cycles were not statistically significantly different across the three hospital types (p = 0.09) nor associated with the pressures achieved during the holding periods (p = 0.29). Some sterilization cycles (Table 3) had holding periods with uneven pressures (pressures intermittently dropping down to lower values).

### Sterile barrier systems

Different sterile barrier systems were used for packaging medical devices for sterilization (Table 4).

**Table 4:** Percentages of reprocessing cycles using different sterile barrier systems for packaging of medical devices.

Sterile barrier system used (n = 189)	Estimate	Standard Error	95% Confidence Interval	
			Lower	Upper
Single wrapped/pouch	35.6%	7.4%	21.2%	53.2%
Double wrapped in wrapping material or pouches, double wrapped container or tray, reusable sterilization container	27.8%	6.0%	16.6%	42.8%
Combination of two or more systems	36.6%	9.6%	18.7%	59.1%

### Effectiveness of autoclave cycles

**Fig 1:**
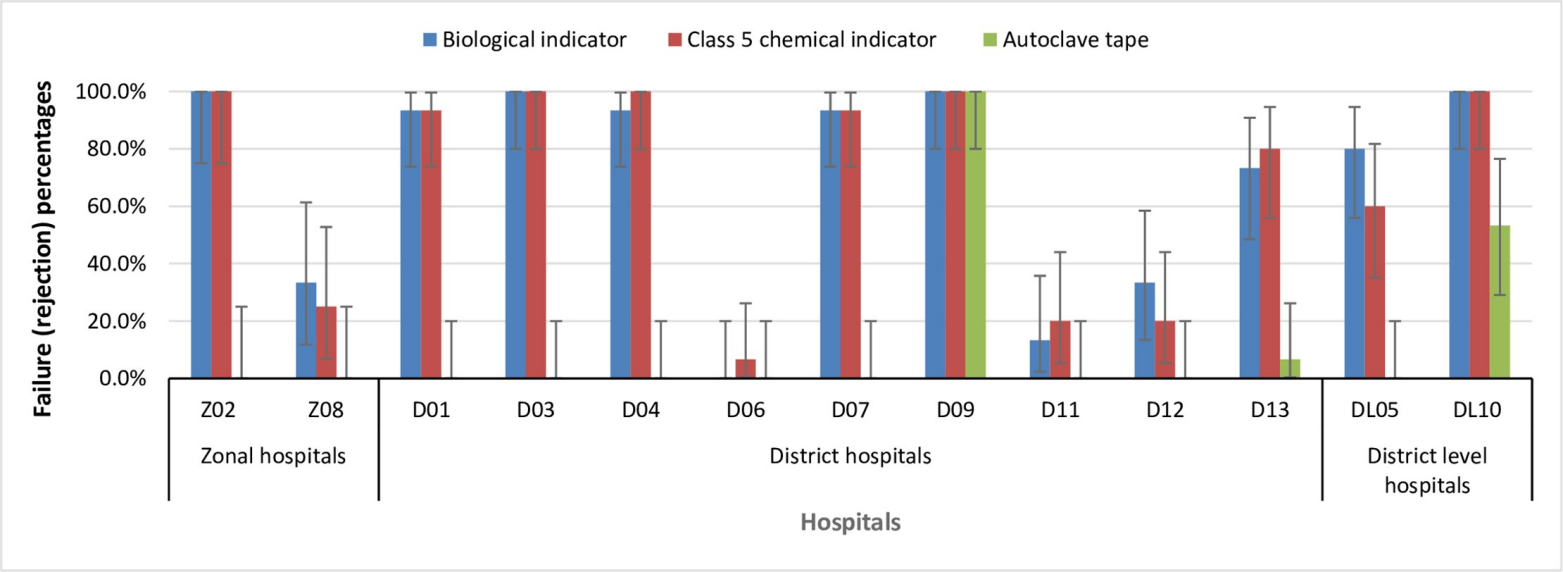
Steam sterilization failure proportions as shown by three different indicators.

The overall proportion of steam sterilization cycles showing positive (ineffective sterilization) results with the biological indicators was 71.0% (95% CI 46.8% - 87.2%). The proportions for different hospital types (Table 5) were not statistically significantly different (p = 0.51). The proportion ranged from 0% to 100% across the thirteen hospitals (Fig 1).

**Table 5:** Results of biological indicators, class 5 chemical indicators and autoclave tape for each hospital type.

Hospital type*	Number of autoclave cycles tested	Estimate	Standard Error	95% Confidence Interval		
				Lower	Upper	
**Proportion of autoclave cycles giving positive results (indicating ineffective sterilization) with biological indicators**						
All hospitals (13)	189	71.0%	9.5%	46.8%		87.2%;
Zonal Hospital (2)	24	66.7%	29.8%	9.1%		97.5%
District Hospital (9)	135	66.7%	12.3%	36.8%		87.3%
District-level Hospital (2)	30	90.0%	9.4%	47.0%		98.9%
**Proportion of autoclave cycles giving ‘rejected’ results (indicating ineffective sterilization) with class 5 chemical indicators**						
All hospitals (13)	189	69.8%	10.1%	44.4%	87.0%	
Zonal Hospital (2)	24	62.5%	33.5%	6.4%	97.6%	
District Hospital (9)	135	68.1%	12.4%	37.6%	88.4%	
District-level Hospital (2)	30	80.0%	18.7%	22.8%	98.2%	
**Proportions of autoclave cycles NOT showing a change in colour of an autoclave tape**						
All hospitals (13)	189	13.5%	8.7%	2.9%	45.1%	
Zonal Hospital (2)	24	0.0%	**	0.0%	12.5%	
District Hospital (9)	135	11.9%	10.2%	1.5%	54.3%	
District-level Hospital (2)	30	26.7%	24.9%	2.1%	86.2%	

* the numbers in parentheses indicate number of hospitals studied

** cannot be calculated

Of the autoclave cycles, 69.8% (95% CI 44.4% - 87.0%) showed ‘reject’ results with class 5 chemical indicators. The rejection proportions across three types of hospitals (Table 5) were not statistically significantly different (p = 0.87). The rejection proportion ranged from 6.7% to 100% across the thirteen hospitals (Fig 1). The biological indicator is usually accepted as the ‘gold standard’ but the sensitivity and specificity of the class 5 chemical indicator was very high (Table 6).

**Table 6:** Cross-tabulation of biological and class 5 chemical indicator test results.

Class 5 chemical indicator		Biological indicator	
		Rejected	Accepted
**Rejected**	Estimate (% within biological indicator)	95.3%	7.4%
	95% Confidence Interval	81.0% - 99.0%	3.3% - 15.7%
	Standard Error	3.1%	2.6%
**Accepted**	Estimate (% within biological indicator)	4.7%	92.6%
	95% Confidence Interval	1.0% - 19.0%	84.3% - 96.7%
	Standard Error	3.1%	2.6%

Overall, 13.5% (95% CI 2.9%– 45.1%) of the sterilization cycles did not show a change in colour of the autoclave tape (black stripes did not appear). The difference in proportions across the three hospital types (Table 5) was not statistically significant (p = 0.62). The results of the autoclave tape were not statistically significantly associated with the results of the biological indicator (p = 0.29) or chemical indicators (p = 0.27).

### Factors associated with ineffectiveness of steam sterilization

For both biological and class 5 chemical indicators, pressure achieved during holding period and autoclave type were statistically significantly associated with steam sterilization failures (positive or rejected) adjusted for holding period, maintenance of pressure and barrier system used (Table 7).

**Table 7:** Complex Samples—Logistic Regression model for sterilization failures.

Predictor Variable	Failure proportion within category	Odds Ratio	95% Confidence Interval	P value
*Model 1*: *Biological indicator result–Positive (ineffective sterilization)*				
Holding period pressure	n = 160			
≥ 15 psi	49.4%	0.02	0.00–0.75	**0.04**
≥ 10 psi to < 15 psi	80.0%	0.03	0.002–0.42	**0.02**
< 10 psi*	95.2%	1.00		
Maintenance of pressure	n = 160			
Continuous	67.5%	0.66	0.16–2.80	0.53
Intermittent*	57.5%	1.00		
Holding period (min)**	n = 160	0.90	0.81–1.00	0.06
Barrier system used	n = 189			
Combination of two or more systems	76.9%	2.49	0.31–19.96	0.35
Double wrapped, double wrapped container or tray, reusable sterilization container	67.8%	2.26	0.87–5.90	0.09
Single wrapped/pouch*	66.1%	1.00		
Autoclave type	n = 189			
Upward displacement (pressure-cooker type)	72.4%	10.33	2.17–49.22	**0.01**
Downward (gravity) displacement*	46.6%	1.00		
*Model 2*: *Class 5 chemical indicator result–Reject (ineffective sterilization)*				
Holding period pressure	n = 160			
≥ 15 psi	49.4%	0.03	0.001–0.87	**0.04**
≥ 10 psi to < 15 psi	76.0%	0.03	0.003–0.31	**0.01**
< 10 psi*	95.2%	1.00		
Maintenance of pressure	n = 160			
Continuous	70.0%	1.67	0.37–7.56	0.46
Intermittent*	45.0%	1.00		
Holding period (min)**	n = 160	0.90	0.80–1.01	0.07
Barrier system used	n = 189			
Combination of two or more systems	78.5%	3.82	0.35–41.59	0.24
Double wrapped, double wrapped container or tray, reusable sterilization container	66.1%	3.45	0.96–12.40	0.06
Single wrapped/pouch*	63.2%	1.00		
Autoclave type	n = 189			
Upward displacement (pressure-cooker type)	71.8%	23.25	5.30–101.95	**< 0.01**
Downward (gravity) displacement*	40.0%	1.00		

* reference category

** continuous variable

## Discussion

Steam sterilization (autoclaving) is the major technique used for sterilizing medical devices in primary and secondary care public hospitals in Nepal. Our study found a high proportion of sterilization failure in these hospitals. Autoclave type and pressure achieved during the holding period of a cycle were statistically significantly associated with such failures. To our knowledge, these failure proportions exceed those reported by previous studies (1.5% to 43.0%) elsewhere [16–30], however, few of these studies used robust methods, and most were conducted in dental care facilities.

The primary endpoint of this study was the globally recommended Sterility Assurance Level (SAL) for reusable medical devices of 10^−6^; the probability of a product remaining non-sterile after exposing it to a sterilization process should be ≤ 10^−6^ using spores of *G*. *stearothermophilus* as the reference standard [36,37]. This means that if an SAL of 10^−6^ is achieved after a sterilization process, one out of 1,000,000 products (each of them containing 1,000,000 spores) would remain non-sterile. Overall, we found that 71% of biological indicators remained non-sterile after exposure to sterilization processes in hospitals in Nepal and there was a similar proportion of failures using class 5 chemical indicators demonstrating this is a robust result. There was considerable variation in failure proportions between the hospitals (Fig 1) leading to a wide 95% CI (46.8% - 87.2%), so the overall failure rate does not necessarily reflect the effectiveness of steam sterilization within individual hospitals. Nevertheless, the high proportion of failure of sterilization cycles demonstrates that an unacceptable number of instruments would be non-sterile following sterilization in the primary and secondary hospitals studied, and it is likely similar results would be found in other institutions in Nepal.

To avoid hampering the daily sterilization activities due to study procedures, biological and class 5 chemical indicators were packaged separately, using the same methods and materials as those used to package the medical devices for an autoclave cycle. While the methods of packaging were the same, this did not exactly simulate the complexities of an actual package containing the medical devices and may have introduced some bias. If anything, this may have led to an overestimate of the effectiveness of the sterilization cycle as the temperature inside a package of medical devices may not come up to the required level as quickly as the temperature within the autoclave chamber [38]. In addition, the autoclave operators, due to the researcher’s presence, and could have operated the autoclave more carefully than usual.

Pressure inside a sterilization chamber should reach 15 psi (above atmospheric pressure) to achieve a sterilization temperature of 121°C. Fewer than half the autoclave cycles reached a pressure of 15 psi or above, which together with use of pressure-cooker type autoclaves, were statistically significantly associated with steam sterilization failure. Neither the holding period, nor evenness of pressure during the holding period, were associated with sterilization failure. The lack of association between time and effectiveness of sterilization cycles is probably related to failure to achieve the required temperature/pressure parameters. In principle, time (holding period) is clearly linked with the effectiveness of sterilization when the required temperature/pressure of the autoclave is achieved [39] so it is essential that all the parameters required for effective sterilization are reached.

A further limitation of the sterilization service was the type of autoclave. Of the 24 autoclaves used in the hospitals, 21 were basic pressure-cooker type autoclaves which have very poor air displacement capabilities [40]. It is recommended that devices sterilized in these autoclaves are used immediately after sterilization [41]. These autoclaves are not appropriate for porous loads, medical devices wrapped in a sterile barrier system, or medical devices having lumens or complex tortuous structures, because they are not effective in displacing air inside such loads or devices with saturated steam. The remaining three autoclaves were gravity displacement autoclaves which are considered superior to pressure-cooker type autoclaves but are also not considered appropriate for these types of medical devices as they also are not very effective in displacing air with steam [42]. We found that reusable medical devices were enclosed within a barrier system for all of the reprocessing cycles. Most of the sterilization loads included porous items and about half of them included items with lumens or tubing. Displacement of dry air from such items and penetration of the steam into them becomes difficult when using non-vacuum pressure-cooker type or gravity displacement autoclaves [43].

The majority of hospitals included in this study did not have dedicated space for reprocessing of medical devices. The number of staff allocated for reprocessing of medical devices was small (i.e. 1 to 3) irrespective of hospital types and sizes. Only two of the hospitals had sterilization procedure manuals or flow charts while only one had spare parts. None of the hospitals had maintenance records for autoclaves and systems for monitoring sterilization processes using chemical or biological indicators. These situations may be indirectly associated with a high proportion of sterilization failures in the hospitals in Nepal.

While it is clear that the steam sterilization processes studied did not guarantee sterility, this does not necessarily mean that the instruments were capable of transmitting infection. Other parts of the process such as disinfection, mechanical scrubbing and cleaning, and desiccation may reduce the microbial load on medical devices and the more susceptible organisms may be inactivated by the sterilization process that was conducted. The risk of transmission of an infectious disease through a contaminated medical device is dependent on additional factors, including the prevalence of the disease in the population and the infectivity of the pathogen [44,45]. Nevertheless, it is incumbent on providers of health care services to minimise the risk to patients, and ensuring effective sterilization of reusable medical devices is a core responsibility.

To ensure safe reuse of medical devices, use of a reliable and affordable process indicator is crucial. We found that high sensitivity and specificity of the class 5 chemical indicators compared with the biological indicators in hospitals in Nepal (p < 0.001). Importantly, in this setting, chemical process indicators are cheaper than biological indicators, and their results are easy to interpret and can be obtained immediately after sterilization. Immediate availability of the results helps when deciding whether or not to release ‘sterile’ devices for immediate surgical use. Therefore, a class 5 chemical indicator could be usefully and effectively employed in hospitals in Nepal to monitor the effectiveness of each steam sterilization cycle. However, only biological indicators can provide the ultimate evidence of effectiveness of a sterilization process, and use a biological indicator at a regular time interval, such as once per week is recommended as part of a quality assurance programme; this approach would ensure the overall effectiveness of the sterilization processes in a hospital [2,5]. If a failed result is obtained with a class 5 chemical or a biological indicator, investigations should be carried out to identify the causes of such failures, and corrective actions need to be taken immediately. Autoclave tape is not intended to determine the effectiveness of a steam sterilization cycle, but to inform healthcare workers whether medical device packages have been exposed to a steam sterilization process by a change in its colour. Also, the findings of this study clearly indicate, as expected, that a change in colour of autoclave tape does not equate to sterility of medical devices and hence, it cannot be used for monitoring the effectiveness of steam sterilization.

To our knowledge, the steam sterilization failure proportion reported by this study for primary and secondary care public hospitals in Nepal is the highest among the steam sterilization failure proportions reported globally. Clearly, there is an urgent need to correct steam sterilization practices in these hospitals, focussing on the sterilization equipment (autoclaves) being used and sterilization temperature/pressure achieved during sterilization cycles. However, all the processes involved in steam sterilization cycles including cleaning, inspection, packaging, sterilization, transport and use should be carried out following standard procedures to ensure the recommended level of sterility of medical devices is achieved and maintained. Management and support processes required for ensuring sterility of medical devices should also be in place. Improving steam sterilization practices in these hospitals could contribute to a reduction in the burden of HAIs in these hospitals and thus save many lives. This study may alert other low- and middle-income countries to possible deficiencies in steam sterilization practices and provide a pathway for identification and improvement of practice and reduction in harm caused by hospitalization.
